# Helping Patients With Persecutory Delusions Find Safety

**DOI:** 10.1093/schbul/sbag034

**Published:** 2026-06-04

**Authors:** Daniel Freeman

**Affiliations:** Department of Experimental Psychology, University of Oxford, Oxford, OX1 3EL, UK; Oxford Health NHS Foundation Trust, Oxford, OX4 4XN, UK

**Keywords:** delusions, persecutory, understanding, treatment

## Abstract

At the heart of a persecutory delusion is an incorrect perception of threat from others. This makes the person feel very unsafe. Overcoming such delusions therefore means establishing a sense of safety, and thus a realisation that there is no current threat. Discovering that one is safe functions as the counterweight to feeling unsafe. The counterweight shifts expectations and changes beliefs. In this article, we describe how safety may be learned. This includes how to engage patients, framing the finding of safety, switching from threat to safety mode, using experiential learning, and consideration of blocking beliefs that may prevent new learning. Key points are to: frame intervention as an opportunity to find greater safety in the world so that meaningful activities can be resumed; repeatedly measure the learning of safety; make new benign associations with the internal and external cues driving the delusions; keep the new learning to the present and future, suitably circumscribed for an individual, so that potential for discord is limited; carry out safety-establishing behavioral experiments that allow for the dropping of defense behaviors, the reframing of evidence for the delusion, and diminish the pervasiveness of the perceived threat; and help patients move from situation-specific instances of safety to more generalised belief change. Enabling patients to re-discover safety in their everyday lives—decisively tipping the scales in favor of the counterweight—is the most transformational element of successful psychological intervention for persecutory delusions.

“O*ut of this nettle danger we pluck this flower safety.*” Shakespeare (Henry IV Part I)^1^

Day-to-day life for patients with persecutory delusions—strongly held, inaccurate threat beliefs that others are trying to harm them—can be exhausting. Thoughts, images, and feelings of attack, humiliation, and catastrophe are uppermost in the mind. The world must be negotiated through this veil of fear. Friends, family, and activities can fall away. Sometimes, the person can feel so defeated that self-care declines. In essence, a person with persecutory delusions feels very unsafe. Hence, overcoming such severe paranoia means feeling safe again. The path to recovery is a process of discovery—the discovery of safety. From our cognitive perspective, a safety belief is the counterweight to a persecutory threat belief.^2^ Rather than dispute the validity of a person’s fears, an alternative position is set out that, with increasing adoption, dwindles the persecutory delusion (i.e., there is less expectation of malign actions from others). But how do we support patients to find safety? In this article, we describe the learning made via the Feeling Safe^3^ and Feeling Safer^4^ cognitive-behavioral treatment programs. These interventions have had successful evaluations with patients diagnosed with psychosis, who, despite prescription of antipsychotic medications, were still experiencing often long-standing persecutory delusions.

## The Theoretical Understanding Underlying the Learning of Safety

For patients with persecutory delusions, certain external stimuli and confusing internal states have become overly associated with fear, threat, and danger. The presence of these stimuli and states drives the sense that something bad will happen. They are seen as the immediate evidence for the persecutory delusion. In the case of external stimuli, the negative association is often with benign or even positive situations, places, and people. It may include, for example, all people, situations in which it is difficult to leave easily such as being in a shop, eye contact, a smile, or use of mobile phones. The world may feel so overwhelming that just leaving the front door becomes associated with threat. Sometimes, bad events have happened in these situations; sometimes, they haven’t. Internally, physiological signs of anxiety and threatening voices commonly, and understandably, take on the associations of threat. Other common internal cues include negative images^5–8^ and dissociation.^7^^,^^9^ Indeed, it is the feeling of threat associated with internal cues that often fosters the negative perception of external stimuli. Feelings of anxiety, or voices warning the person that they are in danger, can lead to an external attribution of threat. So, we must identify the external stimuli and internal cues and help the individual develop new and benign associations. These new associations will counteract the old fear-based ones. Within an inhibitory learning framework of understanding,^10^ the new learning of safety will constrain and dampen the old learning that others intend harm. Therefore, new associations need to be learned, consolidated, and made accessible so that they become the first port of call for understanding. This is the core of successful therapy for persecutory delusions.

The marker of the new learning and the focus of psychological treatment are the person’s beliefs about safety. What needs to be learned is safety with regard to internal feelings and external situations that are genuinely benign. The expectation then becomes one of safety. Threat beliefs come to be seen as outdated. In the language of predictive processing, overly strong high-level priors (probabilistic predictions) become updated.^11^ It is not to say that there is no risk in the world. The assumption is that the individual is largely as safe as most people in everyday situations, albeit that some patients can indeed be more vulnerable.

## Initial Engagement With Psychological Intervention

Our offer to patients is to help them feel safer, happier, and to get back to doing more of the things that they want to do. This is an offering of the counterweights that drive effective therapy. The names of our interventions—Feeling Safe, Feeling Safer—contain the core offer so that it is explicit from the start. This positive offer is what patients need to be willing to engage with in order to take up the intervention. What is *not* required is insight into the presence of a psychiatric disorder or delusions. Patients with absolute conviction in their persecutory delusions can gain large improvements from treatment programs.^12^ We also do not require patients to engage with the terms paranoia or persecutory delusions. They simply need to acknowledge that they experience fears or worries that others are trying to harm them. Then, we set out to understand the evidence for these beliefs while remaining open to the potential for them to be partially or completely correct. This knowledge of the evidence is key to discovering the new associations that must be learned. Typically, we then focus first on getting the person in the right psychological state to be able to discover safety. This means dealing with some of the common maintenance factors of persecutory delusions (eg, worrying, negative self-beliefs, sleep and circadian difficulties).^2^^,^^13–18^

## Framing the Finding of Safety

Most patients with persecutory delusions can agree that their fears have become too dominant. Too much energy is taken up by them, too many activities have fallen by the wayside, and too many social connections are broken. As such, maybe it is time to switch to a different response. Our initial framing of safety is circumscribed. We focus on how things may be now (not in the past) in certain situations and even at certain times of the day. We cannot guarantee complete safety, since there are real dangers in the world. Individuals can experience hostility from others because of, for example, their ethnicity or gender.^19^ What we’re after is the discovery of *sufficient* safety. Maybe it is safe enough now to go to the local shop in the daytime. Maybe it is safe enough to visit family members. Maybe it is safe enough now to do some of the things the person wants to do.

The initial framing of safety is presented in a particular way. There are four parts. First, we describe the person’s current perspective and why it is understandable (eg, “Given what the voices are telling you, and the horrible panicky feelings you get when outside, it makes sense you fear what others may do”). Second, we make clear that this perspective has a cost (eg, “The trouble is that no one seems safe to be around, you are constantly thinking about danger, and you cannot do what you want to do. It must be exhausting.”). We then pivot to a circumscribed safety belief (eg “But perhaps—at least now—there is a little more safety in the world for you. It may be okay to visit the local café, for instance.”). Finally, we highlight the potential benefits of the safety belief (eg, “If it’s true that things may have changed a little, then you could do more of the things you want to do. A little of the weight could come off your shoulders.”). Having identified a potential safety belief, we measure in each session how much it is believed. The safety belief can be refined as the intervention proceeds.

## Switching From Threat to Safety Mode

There are a number of ways to help a person begin to see the world differently. The introduction of the circumscribed safety belief is a key first step. It opens up a flexibility in thinking and a route forward. We can also discuss how safety varies depending on the place, time of day, people present, and our life stage. We can ask how the person would know if things have changed. We can try exercises in which the person imagines both feeling safe and bringing that feeling into the places they want to be. Compassion-focused therapy techniques may help switch modes.^20^^,^^21^ Data logs can be completed where the person notes the signs of safety they’ve spotted in their day. And, importantly, we can begin to introduce alternative explanations for what the person has taken as evidence for the persecutory delusion (eg, “Maybe the problem is really the feelings of anxiety when you are out, and that they aren’t a marker of what others are doing,” “Probably everyone, even you, looks at people when they enter a room, and it doesn’t mean anything more than that,” “Maybe the voices aren’t always right. Maybe it’s best if you decide what is actually going on”). By doing so, we highlight the potential alternative associations that can be learned and embedded through direct experience.

## Finding out From Experience

Direct experiences in the world are crucial to gaining belief in current safety. Such experiences may be ordinary events in the patient’s life (eg, a trip to the shop, a family event) or planned behavioral experiments within the intervention (ie, setting up explicit tests, with predictions of outcomes made beforehand and re-evaluation afterward in light of what actually happened). The experiences and discussion in therapy typically focus on safety in specific situations (eg, safe enough to be on the bus; that there is safety at nighttime). But the aim is to generalize to a more all-encompassing safety belief (eg, “I am okay now to do the things I want to do,” “I am safe in my home now”). Direct experience allows the person to learn new associations for the internal states and external stimuli that they have previously paired with paranoid fears. Where possible, individual cues are separated out and new learning is made about them (for example, focusing on the feeling of physiological arousal without the presence of people and learning that the anxious feelings do not mean that an attack is imminent). Often, however, cues cannot be separated (eg, voices and physiological arousal), so learning must encompass multiple cues.

The primary goal of this work is to find out about safety. It is not set up as an attempt to disprove the delusional belief but rather to evaluate the likelihood of safety. Three delusion maintenance processes are also inherently addressed: the pervasiveness of threat, the immediate evidence for the delusion, and defense behaviors. First, the perceived pervasiveness of threat—the number of potential locations and periods of time in which attacks could happen and hence the number of triggers for paranoia—is gradually reduced. Differentiation is made between people, places, and time of day in the degree of potential risk that they pose. For example, many patients on reflection consider that parents with young children or older adults or some areas of town are less threatening. Second, alternative, more benign explanations for the immediate evidence for the delusion are consolidated (eg, anxious feelings are just anxious feelings and not necessarily a sign of threat from others, what voices say is not always correct). And third, people learn that defenses aren’t necessary. Defenses maintain the delusion via behaviors such as avoidance that prevent the receipt of disconfirmatory evidence or within-feared situation behaviors that prevent the processing of disconfirmatory evidence (eg, “I would have been harmed if I had not left as soon as I did”).^22^ Patients with persecutory delusions can often avoid indoor spaces, outdoor spaces, or interactions with others.^23^ In-situation defenses include precautions in the home (eg, keeping the curtains closed, checking locks), lowering risk when outside (eg, having someone else with them, only going out at certain times of the day), staying vigilant, preparing for escape, and keeping a low profile. For the person truly to discover how safe the world is, they must lower their defences.

Finding out from experience isn’t easy—and this is acknowledged with the patient. Old memories, habits, and gut feelings can be incredibly powerful. Going into feared situations may bring on, for example, feelings of panic or negative voices. The therapeutic art is to choose situations that enable fears to be activated or potentially activated (ie, the right cues are provided) but without provoking so much anxiety that it becomes overwhelming and new learning of safety isn’t made. Attention must also be paid to potential negative post-event processing that could undermine the new learning (eg, worrying what could have happened or the reaction of the perceived persecutors to testing the waters in this way).

## Blocking Beliefs

There can be many reasons why safety is not learned at a particular time. Perhaps levels of anxiety are just too unbearable for the person to believe there is no danger. There may be little else to do but listen to the voices and worry. Or perhaps they find it hard to realize that the battle is over and too many defenses remain in place to feel genuine safety. Another reason can be the specific content of the delusion: blocking beliefs that explain away the non-occurrence of harm. These can function as a cognitive immunization process, a way of rejecting information that does not fit pre-existing beliefs.^24^^,^^25^ For instance, patients may interpret specific instances of safety as a trick by their perpetrators, designed to fool them into lowering their defenses before an attack. They may believe that their persecutors are “playing a long game” of spinning out the emotional torture before the final reckoning. They assume they won’t be directly attacked now but instead at a time of the persecutor’s choosing. Patients may also think they are being “toyed with.” In these instances—tricking the person, playing a long game, being toyed with—devious perpetrators manipulate an uncertain chronology of threat. It’s a combination that allows the person to discount times of safety. These blocking beliefs must be made apparent and the advantages and disadvantages of acting on them reviewed. Alternative viewpoints should be considered, together with a review of their own advantages and disadvantages. The aim is to find a position that allows the person to get back to meaningful activities. Sometimes, the individual undergoes physical harm without anyone being present (eg, feeling that they are being hit, pain in the body) that they ascribe to unusual processes (eg, black magic). These ideas cannot be directly evaluated through behavioral tests nor safety experienced, so the therapeutic focus typically shifts to the reaction to the harm.

## Role of the Therapist

The therapist must first ensure that the patient is in the best possible psychological position to work on discovering safety. There must be a good alliance, clear goals and expectations of therapy, and sufficient attention devoted to reducing the influence of key maintenance factors (eg, worry, negative self-beliefs, sleep disruption). The role of the therapist is multifaceted. Foremost is showing appropriate respect to someone going through the hard process of re-evaluating views. The therapist must model calmness, flexibility in thinking, and reflection; provide information and psychological ideas; help make explicit the different types of evidence driving the delusion and potential alternative explanations for this evidence; and support the person in devising and conducting behavioral experiments centred on finding safety. It can be helpful to provide written or audio accounts from other patients describing their experiences of finding safety. Measurements are taken of how safe the person believes they are. The therapist needs to bring a “let’s find out” attitude and be alongside the person as they learn. They must help the person move from specific instances of safety to more generalized safety beliefs. Importantly, the therapist gently helps the person push forward and be alert to whether the behavioral experiments are too little or too much.

Overall, a positive focus on safety is the driving force of this work to overcome persecutory delusions. Establishment of safety is the fundamental route to create change in expectations of threat. There is no need to directly dispute the delusion, though there can be a helpful reflection on what the finding of safety means for the persecutory ideas. It is also present and future-focused work—typically, there is no need to spend time discussing whether the perceived threats did or did not exist in the past. Initially, the new safety belief coexists with the delusion. Over time, however, the scales can tip decisively in favor of the counterweight. Expectations shift, and views are updated to more appropriate calibrations of the person’s safety in the world. With that change, lives that were on hold can then resume.
